# Conformational and Stability Analysis of SARS-CoV-2 Spike Protein Variants by Molecular Simulation

**DOI:** 10.3390/pathogens14030274

**Published:** 2025-03-12

**Authors:** Gustavo E. Olivos-Ramirez, Luis F. Cofas-Vargas, Tobias Madl, Adolfo B. Poma

**Affiliations:** 1 Department of Biosystems and Soft Matter, Institute of Fundamental Technological Research, Polish Academy of Sciences, ul. Pawińskiego 5B, 02-106 Warsaw, Poland; golivos@ippt.pan.pl (G.E.O.-R.); fcofas@ippt.pan.pl (L.F.C.-V.); 2 Division of Medical Chemistry, Otto Loewi Research Center for Vascular Biology, Immunology and Inflammation, Medical University of Graz, Neue Stiftingtalstraße 6, 8010 Graz, Austria; tobias.madl@medunigraz.at

**Keywords:** molecular dynamics, conformational space, native contact map, probability states, collective variables, protein stability, SARS-CoV-2

## Abstract

We performed a comprehensive structural analysis of the conformational space of several spike (S) protein variants using molecular dynamics (MD) simulations. Specifically, we examined four well-known variants (Delta, BA.1, XBB.1.5, and JN.1) alongside the wild-type (WT) form of SARS-CoV-2. The conformational states of each variant were characterized by analyzing their distributions within a selected space of collective variables (CVs), such as inter-domain distances between the receptor-binding domain (RBD) and the N-terminal domain (NTD). Our primary focus was to identify conformational states relevant to potential structural transitions and to determine the set of native contacts (NCs) that stabilize these conformations. The results reveal that genetically more distant variants, such as XBB.1.5, BA.1, and JN.1, tend to adopt more compact conformational states compared to the WT. Additionally, these variants exhibit novel NC profiles, characterized by an increased number of specific contacts distributed among ionic, polar, and nonpolar residues. We further analyzed the impact of specific mutations, including T478K, N500Y, and Y504H. These mutations not only enhance interactions with the human host receptor but also alter inter-chain stability by introducing additional NCs compared to the WT. Consequently, these mutations may influence the accessibility of certain protein regions to neutralizing antibodies. Overall, these findings contribute to a deeper understanding of the structural and functional variations among S protein variants.

## 1. Introduction

Analysis of the conformational space of proteins is important for understanding their functional properties. To date, critical aspects of the conformational space in proteins ranging from 10 to 80 amino acids in length have been revealed through long-scale molecular dynamic (MD) simulation (on the microsecond to millisecond timescale) [1]. However, MD simulations face certain limitations in complex systems (>100 k atoms) such as the case of the SARS-CoV-2 spike (S) protein. These challenges include not only the required sampling time, but also the determination of well-converged specific features (e.g., distances, angles, etc.) to explain the most relevant events at the macroscopic level [2]. Therefore, a comprehensive and representative conformational sampling of all states is necessary. Only through exhaustive analysis will it be possible to understand the stability of proteins and observe conformational changes that may rarely occur (e.g., slow transitions), yet are directly involved in key biological processes [3]. In this context, novel methods to efficiently analyze long trajectories are implemented; for instance, time-lagged independent component analysis (TICA) [4,5] is often used to deal with long trajectories and, as a result, capture the meta-states in proteins with greater accuracy. However, defining specific features to conduct this analysis can be challenging for single proteins and even more complicated for complex systems. Therefore, the careful selection of these features, also known as collective variables (CVs), is essential to improving predictions of the conformational space of a complex biomolecular system.

The SARS-CoV-2 S protein has been the subject of several biophysical studies in the context of the global COVID-19 pandemic [6,7,8,9]. This is a membrane protein involved in the molecular recognition of the host cell receptor [10]. It forms a homotrimer with two functional subunits: the S1 subunit is responsible for binding to the cell, and the S2 subunit mediates the fusion of the virus with the host cell membrane [11]. The S1 subunit contains the receptor-binding domain (RBD) that interacts with the human angiotensin-converting enzyme 2 (hACE2). This strong and stable binding initiates the virus’ cell entry process. In addition, the S1 subunit presents an N-terminal domain (NTD) which facilitates surface cell adhesion and regulates the prefusion-to-postfusion transition [12]. This protein domain undergoes conformational transitions, particularly between the open (up) and closed (down) state [13,14]. During the COVID-19 pandemic, the SARS-CoV-2 S protein evolved and developed numerous mutations such as substitutions, deletions, and insertions, leading to the emergence of new variants with differing levels of virulence and enhanced transmissibility [15].

Mutations in both the RBD and NTD are critical to study, alongside the induced conformational states associated with them. The Delta variant was the first to become the most prevalent variant [16] in 2020. This variant presents 29 distinct mutations that distinguish it from the Wuhan variant, hereafter known as the wild type (WT) [17], including D614G, L452R, P681R, and T478K with a potential to increase the transmissibility of the virus [18,19]. By mid-November 2021, new variants emerged with an increased transmissibility, among them the BA.1 (Omicron) variant, which became the most prevalent variant worldwide. BA.1 carries an additional 30 mutations, 15 of which are located in the RBD, along with three deletions and three insertions (e.g., ins214EPE) [20]. Some of these mutations (e.g., Y505H, N786K, T95I, N211I, N856K, and V213R) provide a higher positive electrostatic surface potential that improves the affinity for the hACE2 receptor and promote the evasion of antibodies initially developed for the earlier variants [21]. By 2023, XBB.1.5 was identified as an Omicron subvariant resulting from the recombination of two BA.2 sub-lineages, with an important mutation, S486P in the RBD. This variant presented the highest levels of immune escape among previous Omicron sub-lineages [22]. The JN.1 variant emerged by the end of 2023, spreading fast in at least 41 countries [23]. JN.1 was designated a variant of concern due to its higher hACE2-binding affinity and its ability to evade the immune response acquired by vaccines [24]. This variant differs by 39 mutations in the S protein from the previous Omicron XBB.1.5, including the L455S mutation in the RBD, which is known to increase immune evasiveness [25].

Mutations in the S protein modify the behavior of the virus, but more importantly those occurring in the NTD and RBD can affect the protein–protein interaction that may lead to conformational changes. Experimentally, a decreased rate of antibody recognition was observed in the presence of mutations such as C480S/R, E484K/G/D, C488Y/S, and F490L/I/C in the RBD, and Y145D, K150E, and W152R in the NTD [26]. Moreover, the binding affinity and kinetics of the RBD/hACE2 complex in several variants was evaluated by surface plasmon resonance (SPR). The relevant study revealed that only one RBD mutation (N501Y) can increase the binding affinity by ≈10-fold whereas two mutations (K417N/T and E484K) can enhance binding and immune escape [27]. Similarly, single-molecule force spectroscopy by AFM elucidated the role of N501Y in the stability of the hACE2/RBD complex, generating new protein contacts with an energy gain of 10 kJ/mol [28]. The combination of relatively distant mutations in RBD, such as E484K and N501Y, can further increase the RBD/hACE2 interaction 12-fold [29]. The RBD-opening pathway was characterized by weighted ensemble MD, highlighting the role of N343 glycan and the formation of important inter-chain hydrogen bonds (HBs) between T415 of RBD_A_ and K986 of RBD_C_, a salt bridge between R457 of RBD_A_ and D364 of RBD_B_, and a salt bridge between K462 of RBD_A_ and D198 of the N-terminus (NTD_C_) [30]. Such HB contacts are very frequent in MD trajectories, and some are considered highly frequent contacts with 70% presence in the MD simulation. MD simulation was used to capture the nanomechanical stability of the SARS-CoV-2 RBD protein, which was compared to SARS-CoV-1 from 2003 (previous outbreak). The MD study showed a gain of ≈50 pN in mechanical stability for the WT [8]. The effect of mechanical stability in the RBD-nanobody was studied by MD, considering several variants (e.g., Alpha, Delta, and XBB.1.5) in complex with the H11-H4 potent nano-antibody [31]. The computational study shows that earlier variants contributed to the mechanical stability of the complex while later variants (XBB.1.5) exhibited the lowest interface energy. In addition, it highlights the presence of some conserved contacts between the RBD-nanobody (e.g., Y489-Y104, Y489-L105, L482-Y104, and Q493-S103) across several variants [31].

An important aspect that has not been thoroughly investigated is the conformational space of the S protein variants and their assessment through native contact (NC) analysis. Long-equilibrium all-atom MD performed on the microsecond-to-millisecond timescale [32] has revealed that the folding mechanism of proteins engages several NCs. Here, protein stability is defined quantitatively as the persistence of a certain number of high-frequency NCs across the molecular trajectories for a given conformational state in equilibrium. Two limiting cases are expected: the native state maximizes the number of NCs, while the unfolded state minimizes them. Additionally, NC analysis provides essential information for understanding specific molecular interactions that stabilize a given protein conformation in single proteins and their complexes [31]. Furthermore, the distributions of certain CVs allow us to characterize structural differences in the S protein between SARS-CoV-2 variances. Due to experimental limitations, the identification of important NCs is typically approached by MD simulation, which provides atomic-resolution insights. Several computational methods have been developed to extract this information from protein structures, including a distance geometry with a cutoff parameter [33], Q-score [32], the shadow map [34], the Voronoi diagram [35], overlaps (OVs) of enlarged Van der Waals (VdW) spheres [36], contacts of structural units (CSUs) [37], and OVs combined with repulsive CSU (OV+rCSU) [38]; all of these differ in their protocols, but they extract this information from the protein structure. Generally, an NC is determined between non-adjacent residues with sequential distance, i.e., |i−j|≥4 and where the distance between heavy atoms ranges from 4 to 12 Å [38,39]. The use of NCs was initially introduced in Gō-like models [40] and can be currently found in coarse-grained (CG) models like the GōMartini and OLIVES approaches [41,42].

**Figure 1:**
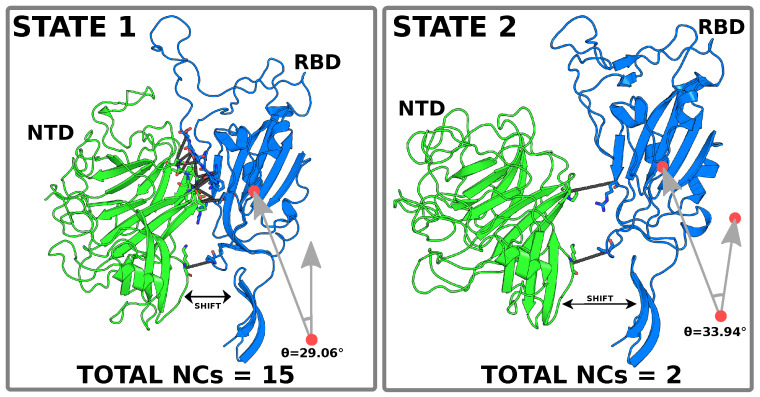
Representation of two states mediated by the formation of protein contacts (shown as solid lines) between the RBD and NTD of the S protein (JN.1 variant, Omicron). The left panel shows 15 NCs between the RBD (blue) and NTD (green), whereas the right panel shows the conformation with only two NCs. In the first case, both protein domains are closer together, while in the second, they are more separated due to the loss of most NCs. This leads to a greater angular fluctuation of the RBD regarding the HR1 domain. The NCs correspond to the set of high-frequency contacts (>60%) identified from the MD simulation.

While most studies have only focused on studying the RBD-ACE2 (protein–receptor) interaction, there is limited knowledge about the intrinsic effect of mutations on the conformational states of the S protein. This is partly due to the complexity of the system and the high computational cost it requires. Furthermore, the flexibility of the trimeric S glycoprotein establishes a number of contacts unique to each macrostate, which remains unclear for most variants of concern. The presence of these contacts and their chemical nature can determine the stability of the structure and its conformational changes. Figure 1 shows two distinct states, each characterized by different NC numbers. Ultimately, understanding these differences may help explain the mechanisms of immune evasion driven by specific mutations. This study determines the role of NCs between the trimeric S protein protomers considering the following variants: WT, Delta, BA.1, JN.1, and XBB.1.5. Through a comprehensive selection of collective variables, we determined the predominant conformational states of the S protein and their corresponding NCs. In addition, we performed an analysis of the chemical character of the most particular states to better understand how certain variants can change the behavior of the S protein interface.

## 2. Materials and Methods

### 2.1. All-Atom Molecular Dynamics Simulation

Trajectories of the WT S protein were obtained from [43,44]. The Amber ff14SB force field [45] and TIP3P water model [46] in a box with a 12 Å padding were used. The system was neutralized with 0.150 M NaCl. The system was minimized using the steepest descent algorithm for 2000 steps, followed by the conjugate gradient algorithm for 3000 steps. Heating was performed in two stages: NVT heating from 0 to 100 K over 50 ps and NPT heating from 100 to 300 K over 100 ps. During minimization and heating, harmonic restraints of 10 kcal mol^−1^ ${Å}^{-2}$ were applied to C-alpha atoms. These restraints were gradually reduced from 10 to 0.1 kcal mol^−1^ ${Å}^{-2}$ during a short simulation of 6 ns at 300 K. Pressure was maintained at 1 atm using the Monte Carlo barostat, whereas temperature was controlled using the Langevin thermostat with a collision frequency of 1 ps^−1^. The Monte Carlo barostat was chosen for its efficient pressure control in the NPT ensemble. Unlike the Berendsen barostat, which does not sample the correct ensemble, or the Parrinello–Rahman barostat (not implemented in AMBER), which can introduce artificial oscillations, the Monte Carlo barostat ensures more stable volume fluctuations. It is widely used in AMBER simulations and has proven effective for water and biomolecular systems [47]. Hydrogen mass repartition was used to enable a 4 fs integration time step [48]. Finally, production was performed for a time of 320 ns with 5 replicas (1.6 μs total). Frames were captured every 0.2 ns resulting in 1600 frames per replica.

Additionally, we performed independent MD simulations for four S protein variants: Delta (PDB ID: 7W92), BA.1 (PDB ID: 7XO5), JN.1 (PDB ID: 8Y5J), and XBB.1.5 (PDB ID: 8VKM), retrieved from the Protein Data Bank [49]. All variants were solved with one RBD in the open (up) state. BA.1 and XBB.1.5 structures were complexed with the hACE2 receptor. For consistency, the hACE2 receptor and all heteroatoms were removed during structure preparation. The Delta variant spanned amino acids 13-1147, BA.1 included residues 26-1146, JN.1 covered 26-1139, and XBB.1.5 ranged 14-1147. Missing residues within these ranges (detailed in Table S2) corresponded to loop regions which were remodeled with Modeller v10.5 [50]. Protonation states correspond to pH 7.4 and were assigned using the PDBFixer [51]. To avoid adding artificial charges, the first and last solved residues were capped with acetyl and N-methyl groups, respectively, to prevent unrealistic interactions and distorted dynamics.

All-atom MD simulations of the four variants were performed with the AMBER22 engine [52] and the FF19SB force field [53], using the pmemd.cuda module for high performance [54]. Each system was solvated in an octahedral box with 20 Å padding using the four-site OPC water model [55]. The systems were minimized using the steepest descent algorithm for 5000 steps. Afterwards, the system was equilibrated using NVT and NPT ensembles. Temperature equilibration was performed in four steps: 150, 200, 250, and 300 K, with each step lasting 200 ps. Harmonic restraints were applied to the heavy atoms of the proteins, with spring constants from 5.0, 4.0, 3.0, and 1 kcal mol^−1^ ${Å}^{-2}$ used in each respective step to allow gradual relaxation. The systems were further equilibrated for 1 ns at 300 K and 1 bar under the NPT ensemble. Production simulations were performed under the NPT ensemble, with five 100 ns replicas for each system, resulting in 500 ns of simulation time per variant. Equilibration and production phases were performed using periodic boundary conditions. Long-range electrostatic interactions were treated with Ewald sums, utilizing a grid spacing of 1.0 Å. Direct electrostatic and Lennard–Jones interactions were both calculated with 9 Å. Hydrogen mass repartitioning with ParmEd [56] enabled a 4 fs integration time step [48]. Temperature control in the NVT ensemble was achieved using Langevin dynamics [57] with a collision frequency of 4.0 ps^−1^, while pressure control in the NPT ensemble employed the Monte Carlo barostat [47] with a 2.0 ps relaxation time. Bond constraints for hydrogens were applied using the SHAKE algorithm [58].

### 2.2. Selection of Collective Variables

**Figure 2:**
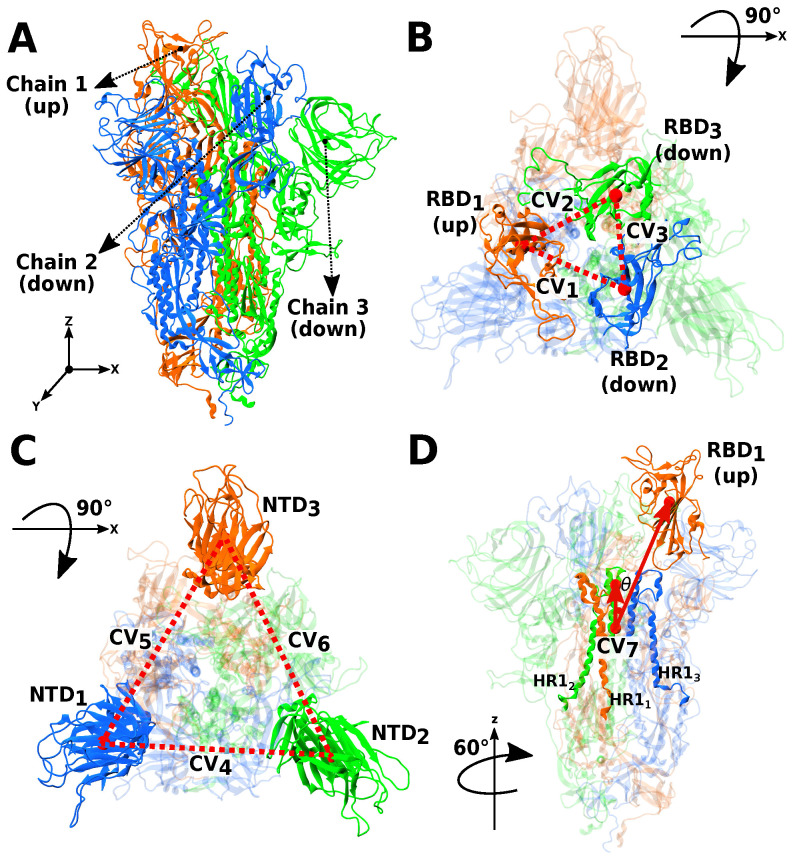
Description of the collective variables (CVs) used to characterize the conformational states of the S protein. Panel (**A**) illustrates the entire system, highlighting one chain with its receptor-binding domain (RBD) in the up state (orange). Panel (**B**) presents CV_1_ to CV_3_, representing the distances between two RBDs (top view). Panel (**C**) depicts CV_4_ to CV_6_, calculated based on the distances between two N-terminal domains (NTDs, top view). Panel (**D**) shows CV_7_, defined as the angle (θ) formed between two vectors: one connecting an RBD and the center of mass of the three HR1 domains (side view). The same definition applies to CV_8_ and CV_9_.

We established nine collective variables (CVs) to determine different conformational states in each system (see Figure 2). The first three CVs were defined by the distances between pairs of RBDs (Figure 2B). For this case, the distances were calculated considering the center of mass of each RBD without counting the most flexible region, the recognition binding motif (RBM, residues 412–480 in the WT model). The next three CVs were established by the distances between the center of mass of each NTD (Figure 2C). In this case, the whole domain sequence was considered to determine the center of mass. Finally, the last three CVs (Figure 2D) were composed of the angle (θ) formed between two vectors: $\vec{v_{1}}$ defined by the center of mass of one RBD and the center of mass of the heptad repeat 1 (HR1) domain of the three chains (residues 912–984) and $\vec{v_{2}}$ defined by the center of mass of the HR1 domain of the three chains and the center of the residues 949–969 of the three chains that are closely aligned to the Z-axis. Angles were determined with Equation (1). A detailed description of the residue set for each CV is given in Table 1.

(1) $$\theta =\cos^{-1}\left(\frac{\vec{v_{1}}\cdot \vec{v_{2}}}{\left|\vec{v_{1}}\right|\left|\vec{v_{2}}\right|}\right)$$

This equation, Equation (1), defines the angle (θ) between two vectors ($\vec{v_{1}}$ and $\vec{v_{2}}$) using the inverse cosine (arccos) function. The expression is derived from the dot product formula, where the numerator represents the scalar product of the two vectors, $\vec{v_{1}}\cdot \vec{v_{2}}$, and the denominator normalizes this product by the magnitudes of the vectors, $\left|\vec{v_{1}}\right|$ and $\left|\vec{v_{2}}\right|$. In our analysis, the magnitudes $\left|\vec{v_{1}}\right|$ and $\left|\vec{v_{2}}\right|$ are defined based on three spatially separated points, corresponding to the center of mass of selected residues in the RBD and HR1 (Table 1 and Figure 2D). As these vectors are always nonzero due to spatial separation, their magnitudes remain strictly positive. This ensures the validity of the equation within our framework, as it prevents division by zero or undefined operations.

### 2.3. Probability Distribution of Conformational States

The probability distribution for each CV was calculated using a histogram for each variant to capture the conformational states of the S protein. A conformational state was considered formed if a peak in the probability distribution of a given CV. In addition, we performed Gaussian fitting as follows:

(2) $$f\left(x\right)=Ae^{-\frac{{\left(x-\mu \right)}^{2}}{2\sigma^{2}}}$$

where μ represents the averages of the Gaussian distribution, σ is the standard deviation, and *A* is the amplitude. This equation remains nonzero, as the distribution f(x) for a given CV is always positive and well-defined in our analysis. When the distributions exhibited two or three conformational states, double or triple Gaussian fitting was applied. The quality of each Gaussian fit was evaluated for statistical accuracy using the $\chi^{2}$ value, calculated with the following formula:

(3) $$\chi^{2}=\sum_{k}\frac{{\left(f_{\mathrm{obs}}^{k}-f_{\mathrm{fit}}^{k}\right)}^{2}}{f_{\mathrm{fit}}^{k}}$$

where ${f^{k}}_{\mathrm{obs}}$ represents the k-th observed value along a CV and ${f^{k}}_{\mathrm{fit}}$ is the corresponding fitted data according to Equation (2) in a given position. In our case, $\chi^{2}$ always returns values larger than zero, ensuring that cases of over-fitting the data are not found.

### 2.4. Calculation of Native Contacts in MD Simulation

We used a combination of structural and chemical-based contact map (CM) determination known as the overlap (OV) and repulsive contacts of structural units (CSUs) approach [38,59]. The OV-CM method determines the presence of an NC between two non-sequential amino acid residues *i* and *j*, if the VdW radii associated with heavy atoms of those two residues show an overlap (i.e., |j−i|≥4). Meanwhile, the rCSU contact map evaluates the chemical properties of the atoms if they are close to each other, adding new contacts to the OV-CM. This approach considers the nature of hydrophobic, hydrophilic, aromatic, ionic, and repulsive (equal charge) residues to define an NC. The NCs were calculated for each state in the Gaussian distributions. Here, we evaluated the MD frames every 100 ps and the NCs were calculated taking all values within the centers of the distributions for each CV (i.e., $\mu \pm \frac{\sigma }{2}$—see Equation (2))—capturing approximately 70% of the data under the curve. We determined the contacts between the RBDs in CV_1_, CV_2_, and CV_3_. For CV_4_ to CV_6_, we determine the NCs between the RBDs and its nearest NTD. For CV_7_ to CV_9_, we determine the NCs between the RBDs and between an RBD and its nearest NTD. The types of interactions between the NCs of each conformational state were distinguished as the following types: ionic, polar, nonpolar, and nonspecific. Ionic interactions were established between NCs presenting one positively (K, R, or H) and one negatively charged (D or E) residue. While polar interactions were established between polar residues (S, T, Y, N Q, or C) and for nonpolar interaction residues of the same type (G, A, V, L, I M, F, W, or P), nonspecific interactions were defined between residues that did not present chemical affinity defined by the OV+rCSU CM. The raw data and scripts used in the analysis are located in an open database [60].

## 3. Results and Discussion

Here, we report the characterization of the conformational space of the S protein within the timescales of AA-MD, mapped using a set of nine CVs. Through the selected CV, we were able to identify distinct conformational states, which, in most cases, were well represented by a Gaussian fit (Table S1). The identification of well-defined states was based on the overlap of the distribution probabilities and the determination of NCs at the protein chain interface. The overall distinction between conformational states in the full-length S protein led to a one-to-one comparison between WT and the other SARS-CoV-2 S variants.

### 3.1. Elucidating the Conformational States Between Two RBDs

**Figure 3:**
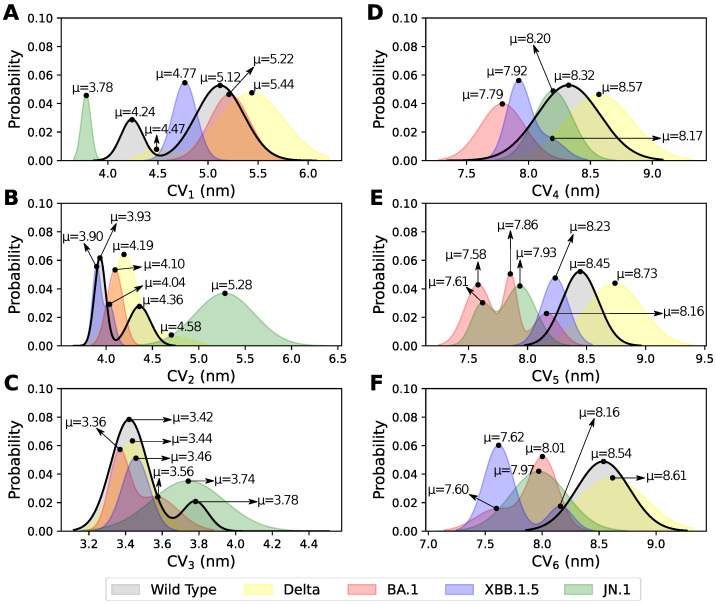
Gaussian distributions of the first 6 CVs based on distances between RBDs (**A**–**C**) and NTDs (**D**–**F**). All CVs are expressed in distances (nm). The distributions were colored for each variant: WT in gray, Delta in yellow, BA.1 in red, JN.1 in green, and XBB.1.5 in blue.

Figure 3A shows the distribution probability for CV_1_, which represents the distance between the center of masses of RBD_1_ and RBD_2_ (see definition in Figure 2B and Table 1). Note that RBD_1_ is in the open conformation (i.e., up state), while the others are in the close conformation (i.e., down state). For WT and Delta variants, the CV_1_ shows two Gaussian distributions whose centers are separated by ≈1 nm. This distance reflects the spatial flexibility between RBD_1_ in the open state and the RBD_2_ in the closed state. To verify the local nature of this interaction, we calculated the presence of NCs at the protein interface. For these two variants, no contacts were found, attributing the molecular flexibility to the associated thermal fluctuations common in MD simulations. In contrast, BA.1, XBB.1.5, and JN.1 variants present one single distribution and thus one single state in our MD simulations. Among these variants, JN.1 distribution occurs at the shortest distances and is well separated from the other variants by ≈1 nm. Notably, XBB.1.5 and JN.1 distributions are among the narrowest, with average σ values of 0.12 nm and 0.05 nm, respectively. In comparison, the WT exhibits σ = 0.24 nm at ${\mathrm{CV}}_{1}=5.12$ nm and σ = 0.11 nm at ${\mathrm{CV}}_{1}=4.25$ nm in WT (see Figures S1, S4, and S5).

On the other hand, the Gaussian distributions of CV_2_ exhibited similar values, ranging from 3.90 nm to 4.60 nm across the WT and the Delta, BA.1, and XBB.1.5 variants. These variants showed a narrower distribution in their states and a smaller separation between their centers, which is ≤0.5 nm (Figure 3B). In this case, the distributions were based on distances between the RBD up (RBD_1_) and the opposite RBD down (RBD_3_) (see Figure 2A). We observed that WT, Delta, and XBB.1.5 variants display two states separated by the formation of specific NCs (see Tables S5, S6, and S8). The WT presented six and four nonspecific NCs that were formed between similar residues (S375, F486, T385, and F456) in both distribution states, respectively, with the center of distribution (μ) at 4.36 and 3.93 nm. Notably, the first state displays two extra specific NCs of nonpolar nature (Table S5). This difference of only two NCs of nonpolar nature suggests a smooth transition between states. In contrast, the NC profile differs greatly in the Delta variant, with 9 and 10 NCs for each state (Table S6). Unlike the WT, more NCs of both polar and nonpolar types are observed. In turn, the XBB.1.5 variant presented a state (μ = 3.90 nm) defined by 24 NCs of ionic, polar, nonpolar, and nonspecific nature. The NCs are reduced to only 14 in the second state of XBB.1.5 (μ = 4.04 nm), losing the two most relevant ionic contacts between D427-H505 and D428-H505 (Table S5). It is interesting to note that in XBB.1.5, the higher number of NCs causes larger stiffness between the RBDs, which is correlated with the shortest fluctuation in the distribution (Table S8). Meanwhile, BA.1 presented 1 nonpolar and 6 nonspecific contacts (Table S7), resulting in a single Gaussian distribution with a slightly greater distance compared to XBB.1.5, which supports the idea that few contacts facilitate conformational transitions. Unlike all variants, JN.1 shows a Gaussian distribution with CV_2_ values spanning larger distances than the other variants, with a difference of >1 nm. In addition, 24 NCs were identified associated with its distribution (Table S9); however, 16 of these NCs are nonspecific, providing less energetic contribution, thus explaining the wide span.

For CV_3_ (Figure 3C), we identified two distinct states in the WT and BA.1. Notably, independent states are formed despite the shorter distances between the two RBDs (down–down). The WT protein presented states with distances of μ = 3.42 and 3.78 nm, which presented 2 and 8 NCs, respectively, most of them nonspecific (Table S5). The Delta variant only presented a single Gaussian distribution (μ = 3.44 nm) with 5 NCs of nonspecific nature similar to WT, but the profile differs greatly (Table S6). In addition, the Delta distribution is within the range of WT, BA.1, and XBB.1.5. Meanwhile, BA.1 exhibited two distributions, separated only by three NCs in the first state and one NC in the second (Table S7). The XBB.1.5 and JN.1 variants only display one state with distances of μ = 3.46 and 3.74 nm, respectively. In XBB.1.5, there are four NCs (one specific and three nonspecific); however, no NCs were observed in JN.1 (Tables S8 and S9). Since JN.1 did not exhibit NCs, its distribution displayed the largest fluctuation among all variants, with σ = 0.20 nm (see Figure 2). For this CV_3_, due to the inherent timescale in AA-MD, some states may be separated by infrequent conformations, which could potentially merge with enhanced sampling techniques.

### 3.2. Conformational States and NCs Identified Between Two NTDs

The two NTDs in the S protein are more distantly separated compared to the two RBDs. Despite this, we observed distinct fluctuations in each variant. Therefore, we analyzed their distributions and NCs using the same approach. Notably, in these cases, the NCs were determined between each NTD and its nearest RBD.

CV_4_ represents the distance between NTD_1_ and NTD_2_ (Figure 2C). Single distributions were observed for most variants, except for XBB.1.5, which exhibited two distributions (Figure 3D). The individual distributions were broader and well differentiated among the variants. In addition, these distributions exhibit few NCs as the NTD evaluated here is the closest to the RBD up conformation. For instance, WT (μ = 8.32 nm) presented only four nonspecific NCs and BA.1 (μ = 7.79 nm) presented two NCs (one nonspecific and one nonpolar) (Tables S10 and S12). No NC formation was observed in the Delta and JN.1 variants for CV_4_ (Tables S11 and S14). In XBB.1.5, we identified one state (μ = 7.92 nm) with 10 NCs, and the second state (μ = 8.17 nm) with 12 NCs (Table S13), with similar proportions between specific and nonspecific NCs. Interestingly, the Delta and JN.1 variants have distributions close to WT, separated by approximately ± 0.2 nm. In contrast, BA.1 and XBB.1.5 are more separated in relation to the WT (≈0.5 nm) and presented the shortest distances (Figure 3D). This suggests a greater compactness within these domains (i.e., RBD and NTD) for these variants that are also more distant from WT in terms of genetic variability (see Figure S6).

In contrast, in CV_5_, we observed Gaussian distributions that are more spread out regarding CV_4_ (Figure 3E). In terms of NCs, we observed a higher number of contacts in variants that are distant. WT, Delta, and XBB.1.5 presented single distributions and these were close to each other, with centers separated by ≈0.3 nm. These three distributions were also rigid as the σ values ranged between 0.11 and 0.24 nm (see Figures S1, S2, and S5). In addition, the NCs of these three variants are mostly nonspecific, with R357 and P521 being relevant in these three profiles (Tables S10, S11, and S13). Conversely, BA.1 and JN.1 presented more than one Gaussian distribution, but with smaller distances compared to the previous ones. BA.1 presented up to three different distributions with NC profiles that varied significantly, with 21, 26, and 4 NCs for each state (Table S12). The two states with the shortest distance presented one ionic contact (R357-E167), which contributed to stronger binding between these domains. Additionally, a dominance of NCs involving positively charged residues was observed, including R355, R357, R466, H519, and K114. However, the third state only presented four nonspecific NCs. It is noteworthy that ionic contacts have been rarely observed at the S protein interface in previous variants (WT and Delta). In addition, the distribution of the JN.1 is within the range of BA.1, but with only two states, presenting 14 and 13 NCs, respectively, most of them nonspecific (Table S14).

The distances observed in CV_6_ were smaller, suggesting a closer relationship between associated the NRD of the RBD down (Figure 3F). In this case, we observed that WT and Delta exhibited single distributions with their center at greater distances. The wide distribution of these variants is driven by the lack of contacts, only one in WT (P230-N394) and three in Delta (R357-P230, A520-K41, and P521-K41) (Tables S10 and S11). The BA.1, XBB.1.5, and JN.1 variants had smaller distributions similar to each other. BA.1 has two states with seven and six NCs, respectively. Notably, only one NC (Q116-I468) distinguished two states, but due to the AA-MD timescale, it may be required to extend the simulation or number of replicas to determine the statistical significance of the two-state model. In contrast, JN.1 exhibited a single distribution, dominated by ten NCs, one polar and nine nonspecific (Table S14). In the case of XBB.1.5, we found a greater separation and also a greater difference in the NC profile of each state. One of the states (μ = 7.62 nm) is dominated by two polar and six nonspecific NCs; meanwhile, the second (μ = 8.16 nm) is determined by the same two polar contacts and twelve other nonspecific NCs (Table S13). These results suggest that most states are determined by nonspecific contacts, a trend observed across the previously evaluated CVs. However, the presence of a few ionic, polar, or nonpolar NCs can contribute to the formation of distinct states. In the case of Omicron variants (e.g., BA.1 and XBB.1.5), these states are more compact compared to the WT. In the next section, we will introduce a different CV to analyze large conformational changes based on the angle between the RBD-HR1 subunits.

### 3.3. Conformational States and NCs Identified with Angle Between RBD-HR1 Subunits

The CV defined as an angle is a useful measure to evaluate fluctuations of large domains [61]. Here, we analyzed the distribution of angles formed between two specific vectors (Figure 2D), which reflects the flexibility of each RBD (Figure 2D). Likewise, for each case, we determined the occurrence of NCs between the RBDs and between one RBD and its nearest NTD unit.

**Figure 4:**
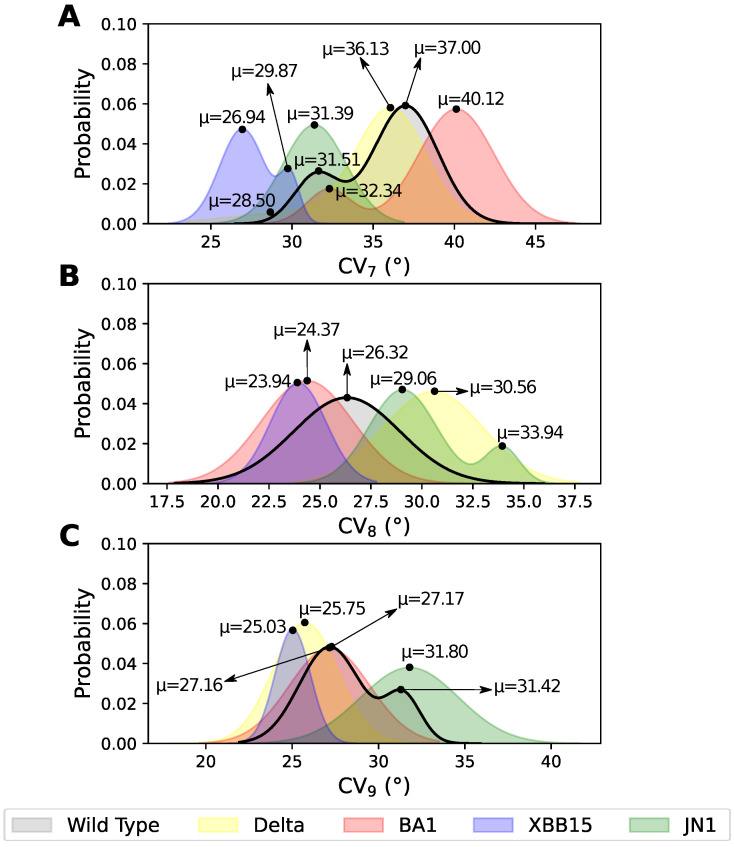
Gaussian distribution of the CVs defined by the angle formed between two specific vectors involving RBD_1_ up (chain-**A**), RBD_2_ down (chain-**B**), and RBD_3_ down (chain-**C**). The angle is measured in degrees (°). The distributions follow the same color pattern for each SARS-CoV-2 variant.

CV_7_ exhibited the largest angular fluctuation as it involves the RBD up (Figure 4A). Across all variants, no NCs were identified between the RBD up and its opposite RBD, consistent with CV_1_ data indicating the absence of NCs. Contacts between the RBD and its closest NTD were mainly observed here. The WT and Delta variant display similar distributions, with two states separated by ≈6°. However, these states were clearly distinct, as Delta did not present NCs between the RBD and the NTD (Table S16). This is also in agreement with CV_4_. Therefore, we can infer that Delta variability, particularly the T478K mutation in the RBD, induces a greater separation between these domains. The BA.1 variant is also close to WT in terms of Gaussian distribution (Figure 4A), but with a fluctuation of ≈3°. The NCs identified in BA.1 are large in number and mostly specific ones (Table S17) in the state close to the WT (μ = 32.34°). It is interesting to note the presence of two ionic contacts, R357-E167 and R466-E133, that provide better stability to those domains. In contrast, the second state (μ = 40.12°) lost all its NCs, retaining only one nonpolar NC, in agreement with its intrinsic flexibility. On the other hand, JN.1 and XBB.1.5 present distributions with lower angular values than the WT, which denotes increased rigidity. XBB.1.5 exhibits the most compact states due to the greater number of specific NCs between the domains. Here, we observe two states with similar NC profile and similar ratio between specific and nonspecific NCs (Figure 4A and Table S18). In contrast, JN.1 (Table S19) did not present NCs between these domains (i.e., NTD and RBD), similar to Delta. Therefore, its compactness is likely not determined by NCs but by structural changes in other domains.

Similarly, CV_8_, which measures the flexibility of RBD_2_ (in down conformation), has a higher compactness in all variants denoted by the lower values compared to CV_7_, ranging from ≈24° to 34° (Figure 4B). All variants except JN.1 exhibited a single state with a broad Gaussian distribution. The WT exhibits the highest flexibility, denoted by the σ value of 2.61 (see Figure S1). In contrast, the variants had a greater compactness, with lower σ values related to a significant presence of NCs (Tables S16–S19). Interestingly, with CV_8_, we observed NCs between both RBDs and between RBD and NTD units. We also noticed a large number of specific NCs between RBDs, the Delta variant with five specific NCs (Table S16), BA.1 with four specific NCs, among them an ionic D428-H505 arising from the Y505H mutation (Table S14), JN.1 with eight and thirteen specific NCs in its two states, respectively (Table S19), and XBB.1.5 with seven specific NCs of which two were ionic caused by the Y505H mutation similarly (Table S18). A consistent trend was observed: WT had fewer contacts, while the four variants displayed a higher number of NCs, most of which were specific (forming ionic, polar, and nonpolar interactions). Additionally, the JN.1 variant exhibited the highest number of contacts, with up to 36 NCs, most of which were nonspecific. This higher number of NCs indicates a marked difference for the RBD_2_ of this particular variant, in agreement with the different distribution (Figure 4B).

Finally, CV_9_ measures the flexibility of the RBD_3_ (in down conformation). In this case, the WT is defined by two states, while all other variants displayed single distributions, ranging from ≈25 to 32° (Figure 4C). This RBD exhibited a fluctuation range similar to the opposite RBD, which was also in the down conformation (≈20° to 35°, Figure 4C). Therefore, we can infer that their dynamics in terms of protein stability are highly similar. In addition, the Gaussian distributions of all systems are closer to each other, with a maximum deviation of ≈4° (Figure 4C). The states of WT are defined by mostly nonspecific NCs, six between RBDs and up to five between the RBD and NTD (Table S15). In Delta and BA.1 variants, we observed a similar number of contacts between the RBDs (four and one NCs, respectively) and NTDs (one and four NCs, respectively) (Tables S16 and S17). XBB.1.5 has NCs in both cases, between RBDs (three NCs) and between an RBD and NTD (nine NCs) (Table S18). The JN.1 variant displayed fewer NCs overall (Table S19), with no NCs between RBDs but ten NCs between RBD and NTD units. Additionally, we observed that JN.1 exhibited a markedly different profile between RBD_2_ (CV_8_) and RBD_3_, with numerous contacts around RBD_2_ and none around RBD_3_.

### 3.4. Role of the Mutations on the Conformational States of the S Protein

We identified the formation of specific NCs in each state, considering the conserved and non-conserved residues. The NCs that occur in a conserved manner (with non-mutated residues) predominate between the RBD and NTD subunits, suggesting that surface stability between these two domains is maintained throughout the virus’s evolution. This is consistent with the greater conservation observed near the stalk of the S protein [62]. In contrast, the NC profile for each state varied significantly between RBDs, with greater differences observed in variants with more distant sequences.

**Figure 5:**
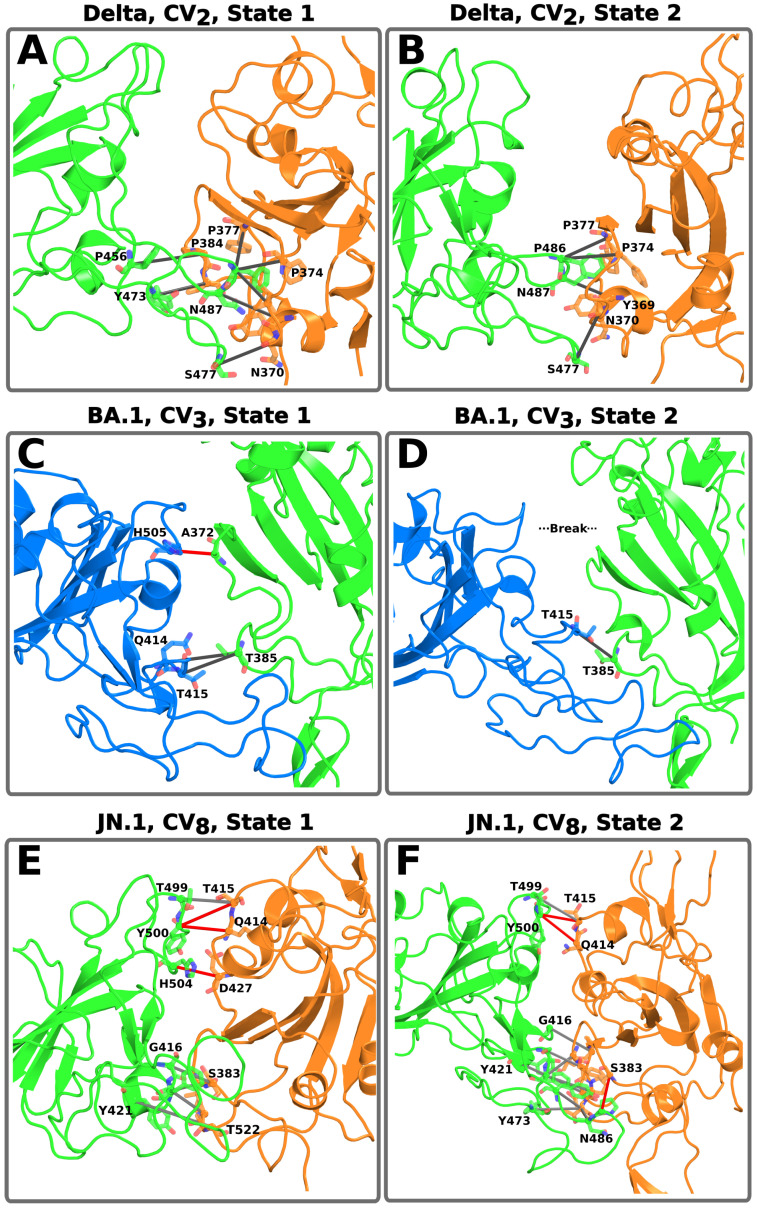
Formation of specific NC among the RBDs, mediated by conserved (gray dashes) and mutated (red dashes) residues. Panel (**A**,**B**) show the RBDs (CV_2_) of the Delta variant. Panel (**C**) displays how mutation Y505H, associated with the BA.1 variant, leads to a new NCs in the distal part of the RBD, which were lost in the second state (**D**). Panel (**E**,**F**) demonstrate that in the JN.1 variant, multiple mutations (red dashes) lead to the formation of new NCs in both states, resulting in RBD compaction. RBD colors follow the same definitions from Figure 2.

In the Delta variant, the mutation T478K drives the formation of three new NCs, as calculated with CV_2_ (Figure 5A,B), which were not observed in the WT. Notably, T478K is located in the RBM region and is present in all the investigated variants (Figure S6). These new NCs are formed with conserved residues in the opposite chain, such as N370, S371, and A372 (Table S6). Furthermore, this group of new NCs occurs only in one state of CV_2_, leading to a different state with greater distance between the RBDs. The T478K mutation has been listed as an important modification for altering the interface potential of the RBD by making it more positive. However, there are conflicting reports on whether this mutation enhances the specific interaction with the hACE2 receptor [63]. Additionally, no studies have reported that T478K improves H-bond or salt bridge formation with hACE2, but an improvement in electrostatic forces of 2.8 kcal/mol was reported in [64]. We point out that T478K does not induce the formation of new NCs in the following variants, and therefore, its role is limited to this variant.

In contrast, the BA.1 variant exhibited fewer NCs resulting from RBD mutations (Figure 5A,B). Among these, only the Y505H mutation generated a new specific interaction with D428. This mutation is particularly significant due to its ionic nature, which substantially enhances interactions between RBDs. The formation of this new NC was identified in both CV_2_ and CV_8_ (Tables S7 and S17). Previous studies have reported that YH505 affects the function of the S protein by decreasing its affinity for hACE2 [21,65,66]. In that sense, our results suggest that Y505H would stabilize the RBDs and make the interface more compact, at the cost of decreasing the affinity for hACE2. This observation aligns with reports indicating that this mutation increases the potential for antibody blockade [67]. Given this information, we propose that the BA.1 variant may exhibit a reduced accessible surface area at the protein interface, which could serve as an efficient mechanism to evade neutralizing antibodies.

In the case of XBB.1.5, we also identified new NCs attributed to mutations only among RBDs, in particular for CV_2_, CV_3_, CV_8_, and CV_9_ (Tables S8 and S18). The behavior of the RBDs in this variant is highly similar, as are the NCs attributed to specific mutations. This suggests that evaluating the same domain using different CV types (e.g., distance- and angle-based) may be beneficial for validating the impact of key mutations. In CV_2_, three mutations (S373P, F486P, and Y505H) are associated with the formation of three NCs of specific nature (P373-P486, D427-H505, and D428-H505), while four mutations (S373P, K417N, Q498R, and N501Y) lead to five new nonspecific NCs (P373-N487, G381-N417, G413-R498, Q414-R498, and D427-Y501). CV_3_ is less influenced by mutations as it presents few contacts; only D405N and S375F formed three new nonspecific NCs (N405-F374, N405-F375, H505-F375) (Table S8). In CV_8_, we found three mutations (S373P, F486P, and Y505H) that lead to the formation of four new specific contacts (P373-P486, F374-P486, D427-H505, and D428-H505), two of them ionic. Likewise, mutations Q498R and N501Y give rise to four nonspecific NCs (G413-R498, G413 Y501, Q414 R498, and D427 Y501) for CV_8_. For CV_9_, we detected that the D405N mutation generates two new nonspecific NCs (N405-F374 and N405-F375). In agreement with the above, there are no NCs related to mutated residues in this variant (Table S18). Notably, in some cases, a single mutation can lead to the formation of multiple NCs, as seen with Y505H, which generates two new NCs.

The JN.1 was the variant with the highest number of mutated residues, resulting in a distinct NC profile. Specifically, with CV_8_, we identified the highest number of NCs among two RBDs (RBD_1_ and RBD_3_), which are the closest to each other. Both of these states have new NCs attributed to mutations (Figure 5E,F). In the first state, the N500Y and Y504H mutations give rise to new NCs of polar and ionic nature, respectively. Other mutations of interest are N405, N417, S455, R497, and H504, although these only generate new nonspecific NCs. Notably, the R497 and H504 mutations increase the positive charge of the RBD surface, altering the domain’s electrostatic potential, a mechanism that enhances antibody evasion, particularly in Omicron variants [68]. On the other hand, the second state has a greater number of mutations that generate new NCs. Among these, F485P, D405N, K417N, G455S, T483K, F485P, N500Y, and Y504H primarily interacted with conserved residues on the opposite chain (Table S19). This demonstrates that the S protein (Delta, BA.1, XBB.1.5, and finally JN.1) is moving towards a structure with a higher number of NCs, which makes the RBDs more compact and grants a high potential for immune evasion.

## 4. Conclusions and Perspectives

Several MD studies have focused on determining the effect of mutations on RBD/hACE2 complex stability. In this study, using AA-MD simulation and contact map analysis, we reveal the key role of mutations in shaping different conformational states of the S protein and quantify the associated NC profiles. Through statistical analysis based on the distribution probabilities of distance- and angle-based CVs, we demonstrated that several variants exhibit a strong tendency to restrict RBD motion. This tendency is more marked in Omicron variants (BA.1, XBB.1.5, and JN.1). Furthermore, we determined that mutations impact interactions between RBDs more than between the RBD and NTD. Additionally, our computational analysis highlights the importance of specific mutations in defining distinct conformational sates in the S protein, beyond their well-established role in enhancing RBD/hACE2 interactions. In particular, T478K, N500Y, and Y504H are found to display this importance. Here, we have observed that these mutations also originate new NC pairs that determine different conformational states. Moreover, the NC profiles provide valuable insights into protein stability and facilitate comparative analyses of the conformational space across several SARS-CoV-2 S variants.

In addition, it is important to emphasize that the transitions between the up and down states are inherently stochastic and typically occur on longer timescales. Several studies have employed enhanced sampling techniques to characterize these transitions [14,30,69,70]. While these approaches (biased MD) extend accessible timescales, they may also introduce unphysical transition pathways. Therefore, to capture more realistic transition states, unbiased simulations are generally necessary. This is not only necessary to characterize the open and closed states, but also for the transition from prefusion to postfusion states and the effect of mutation for those cases. Although we performed extensive conformational sampling (500 ns) using unbiased MD of the full trimeric structure, this approach may still limit the observation of long-timescale conformational changes. However, our conformational sampling effectively captures fluctuations around the initial state (up conformation), allowing for a comparative analysis of the effect of mutations. Accessing timescales in the micro- or millisecond range is only possible with high-performance computational infrastructures, such as that realized in the aerosol virus simulation [71]. This limitation can be overcome in future studies through coarse-grained methods, which extend accessible timescales by one or two orders of magnitude. In this line, the characterized conformational states and NC profiles can further aid the development of effective CG models, enabling the exploration of larger length scales (e.g., virion level) and longer timescales (e.g., sub-millisecond), thereby bringing simulations closer to biological conditions.

Finally, our findings are consistent with both experimental [72,73,74] and computational studies [75]. As mentioned, the most recent variants (Omicron, particularly JN.1) tend to adopt a more compact conformational state in the interphase (near the RBD and NTD), accompanied by the formation of new NCs between mutated–mutated or mutated–conserved residues (e.g., T478K, N500Y, and Y504H). From this, we infer that the energy barrier for the transition between the open and closed states may be higher, which could affect the overall fitness of the virus. However, this structural alteration also confers an advantage in immune evasion, both for innate and acquired immunity (such as that induced by vaccination), as recently reported [76]. This involves reduced accessibility of critical epitopes, which could hinder recognition by neutralizing antibodies, contributing to immune escape. Although the stability of the RBDs is altered in the new variants, they remain functional for binding to host cell receptors, leading to increased viral infectivity. This reflects a balance between reduced hACE2 affinity and enhanced immune evasion, as suggested in other studies [77]. These changes in the dynamics of the protein underscore the adaptive mechanisms of the virus and suggest that variants with such structural modifications may exhibit both enhanced immune resistance and increased transmissibility.

## Figures and Tables

**Table 1:** Definitions of the range of residues for the 9 collective variables (CVs) employed in this study. The type 1 CV involves the RBD, type 2 the NTD, and type 3 is defined between the RBD and center of mass (COM) of HR1.

CV	Measure	Index	Range of Residues Included in COM Calculations	
Type 1	*d*	1–3	RBD_1_ (up): I338-W442 to Q512-G532	RBD_2_ (down): I338-W442 to Q512-G532
Type 2	*d*	4–6	NTD_1_: A31 to F311	NTD_2_: A31 to F311
Type 3	angle (°)	7–9	$\vec{v_{1}}$ = RBD_1_ (up): I338-G532 and HR1_1_– HR1_3_: L943-Q1015	$\vec{v_{2}}$ = HR1_1_–HR1_3_: L943-Q1015 and HR1_1_–HR1_3_: S980-R1000

Note: *d* = distance in nm and (°) angle in degrees.

## Data Availability

The original data presented in the study are openly available at https://doi.org/10.5281/zenodo.14793235, accessed on 20 January 2025.

## Supplementary Materials

The following supporting information can be downloaded at the following link: https://www.mdpi.com/article/10.3390/pathogens14030274/s1; Figure S1: Gaussian distribution of the WT protein for all calculated CVs.; Figure S2: Gaussian distribution of the Delta variant for all calculated CVs.; Figure S3: Gaussian distribution of the BA.1 variant for all calculated CVs.; Figure S4: Gaussian distribution of the XBB.1.5 variant for all calculated CVs.; Figure S5: Gaussian distribution of the JN.1 variant for all calculated CVs.; Figure S6: Alignment of the primary sequences of the WT S protein and its 4 variants.; Table S1: Values of each CV (calculated between RBDs), σ, and $\chi^{2}$ averages of the different states obtained by the probability distribution.; Table S2: Values of each CV (calculated between NTDs), σ, and $\chi^{2}$ averages of the different states obtained by the probability distribution.; Table S3: Values of each CV (calculated with angles of RBDs), σ, and $\chi^{2}$ averages of the different states obtained by the probability distribution.; Table S4: Missing residues range remodeled into the chains of the S protein variants.; Table S5: High frequency NCs (freq > 60%) between the RBDs in the WT protein.; Table S6: High frequency NCs (freq > 60%) between the RBDs in the Delta variant.; Table S7: High frequency NCs (freq > 60%) between the RBDs in the BA.1 variant.; Table S8: High frequency NCs (freq > 60%) between the RBDs in the XBB.1.5 variant.; Table S9: High frequency NCs (freq > 60%) between the RBDs in the JN.1 variant.; Table S10: High frequency NCs (freq > 60%) between an NTD and RBD in the WT protein.; Table S11: High frequency NCs (freq > 60%) between an NTD and RBD in the Delta variant.; Table S12: High frequency NCs (freq > 60%) between an NTD and RBD in the BA.1 variant.; Table S13: High frequency NCs (freq > 60%) between an NTD and RBD in the XBB.1.5 variant.; Table S14: High frequency NCs (freq > 60%) between an NTD and RBD in the JN.1 variant.; Table S15: High frequency NCs (freq > 60%) for the CVs calculated with angles in the WT protein.; Table S16: High frequency NCs (freq > 60%) for the CVs calculated with angles in the Delta variant.; Table S17: High frequency NCs (freq > 60%) for the CVs calculated with angles in the BA.1 variant.; Table S18: High frequency NCs (freq > 60%) for the CVs calculated with angles in the XBB.1.5 variant.; Table S19: High frequency NCs (freq > 60%) for the CVs calculated with angles in the JN.1 variant.
