# Supplementary material for: Conformational and Stability Analysis of SARS-CoV-2 Spike Protein Variants by Molecular Simulation
Source: Pathogens. 2025 Mar 12;14(3):274. doi: 10.3390/pathogens14030274 (PMC11945020; doi:10.3390/pathogens14030274)
Supplement: Supplementary file 1 [file pathogens-14-00274-s001.zip › pathogens-3502314-supplementary.pdf]

Conformational and stability analysis of SARS-CoV-2 spike protein variants by molecular simulation

Gustavo E. Olivos-Ramirez, Luis F. Cofas-Vargas, Tobias Madl, Adolfo B. Poma

Supplementary Figures

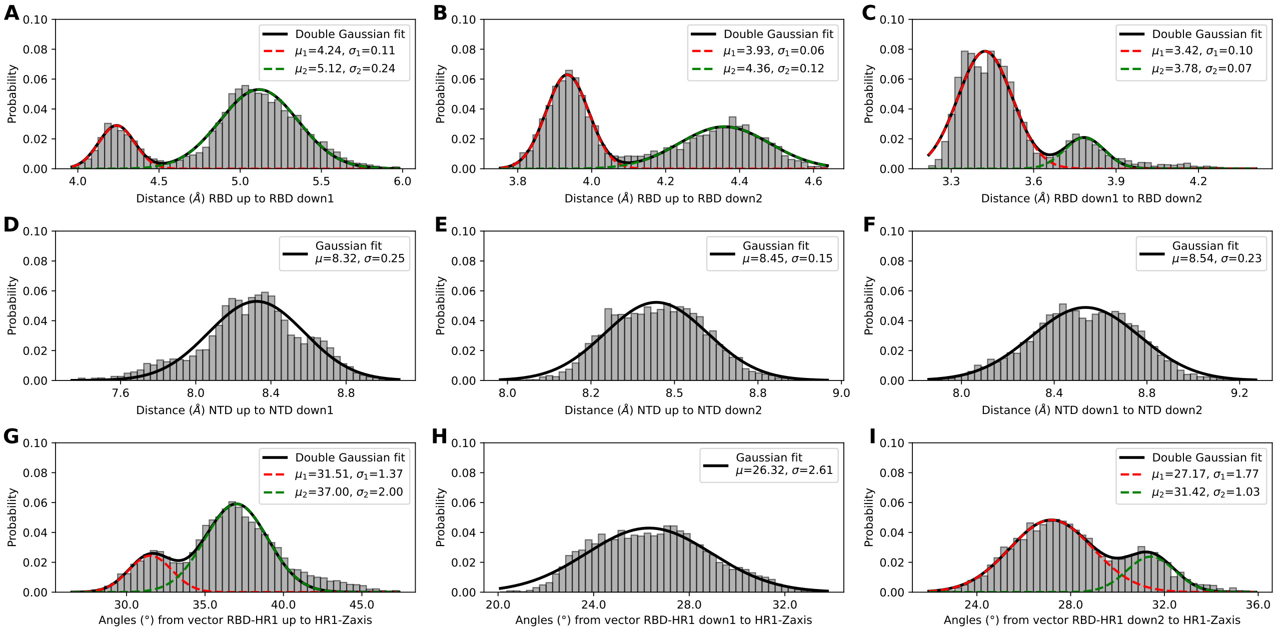

Figure S1: Gaussian distribution of the WT protein for all calculated CVs. The black line indicates a fitting for a single Gaussian distribution while the dotted lines (red or green) for a double distribution.

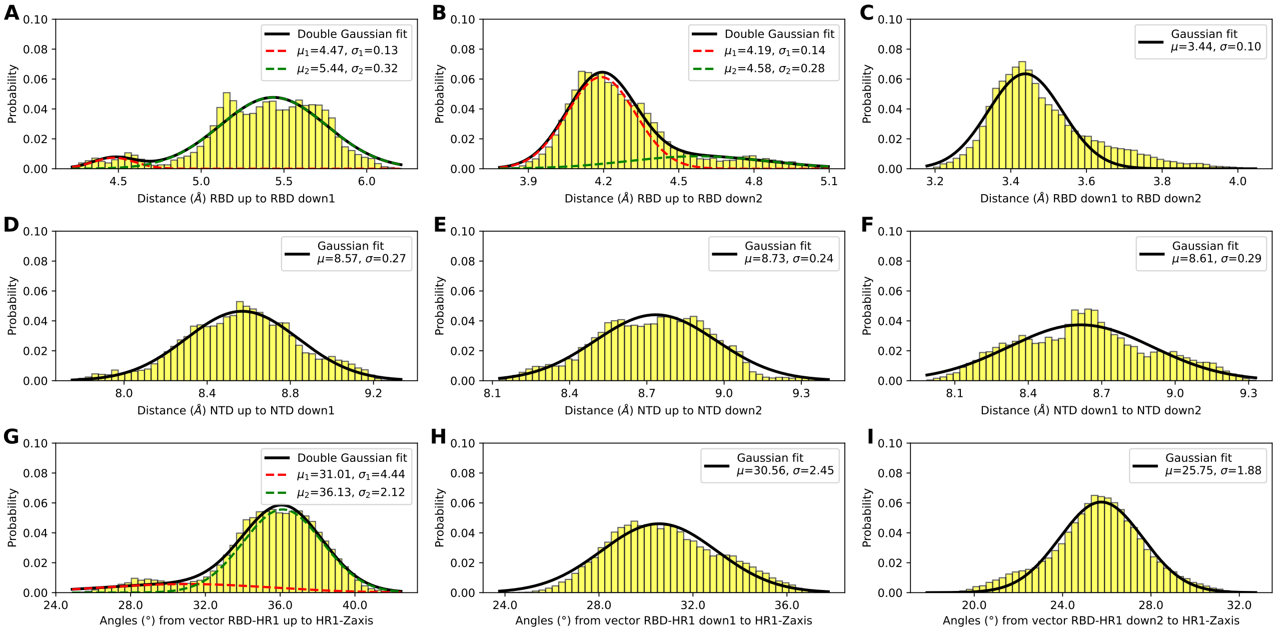

Figure S2: Gaussian distribution of the Delta variant for all calculated CVs. The black line indicates a fitting for a single Gaussian distribution while the dotted lines (red or green) for a double distribution.

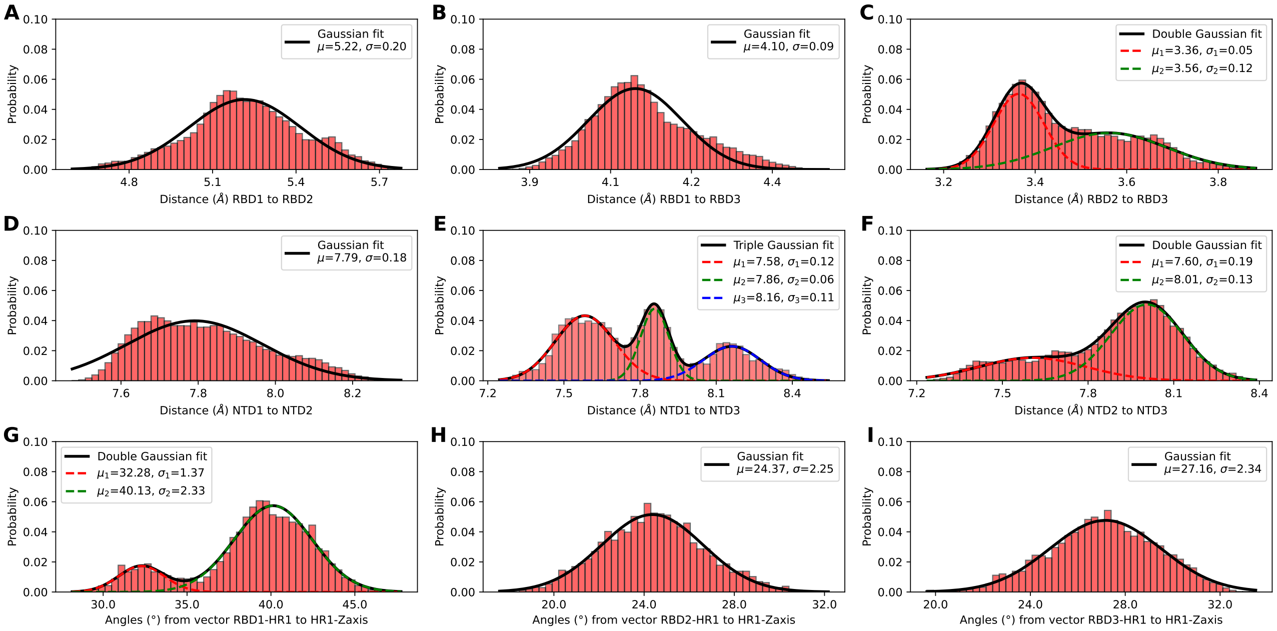

Figure S3: Gaussian distribution of the BA.1 variant for all calculated CVs. The black line indicates a fitting for a single Gaussian distribution while the dotted lines (red, green, or blue) for a double or triple distribution.

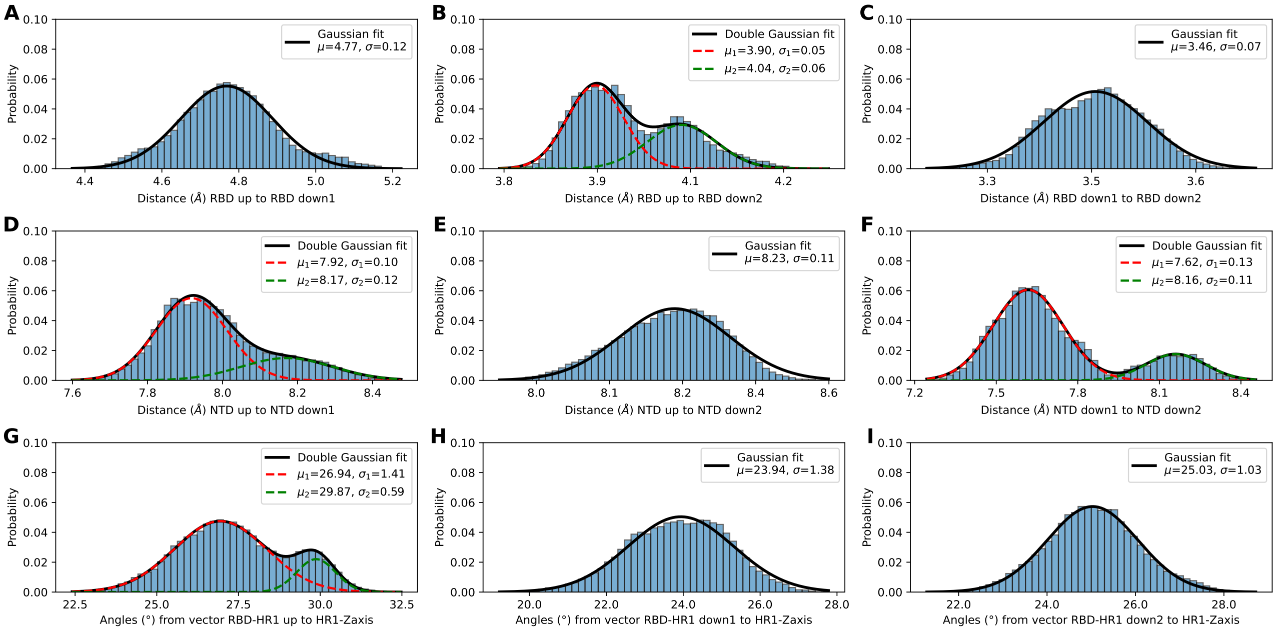

Figure S4: Gaussian distribution of the XBB.1.5 variant for all calculated CVs. The black line indicates a fitting for a single Gaussian distribution while the dotted lines (red or green) for a double distribution.

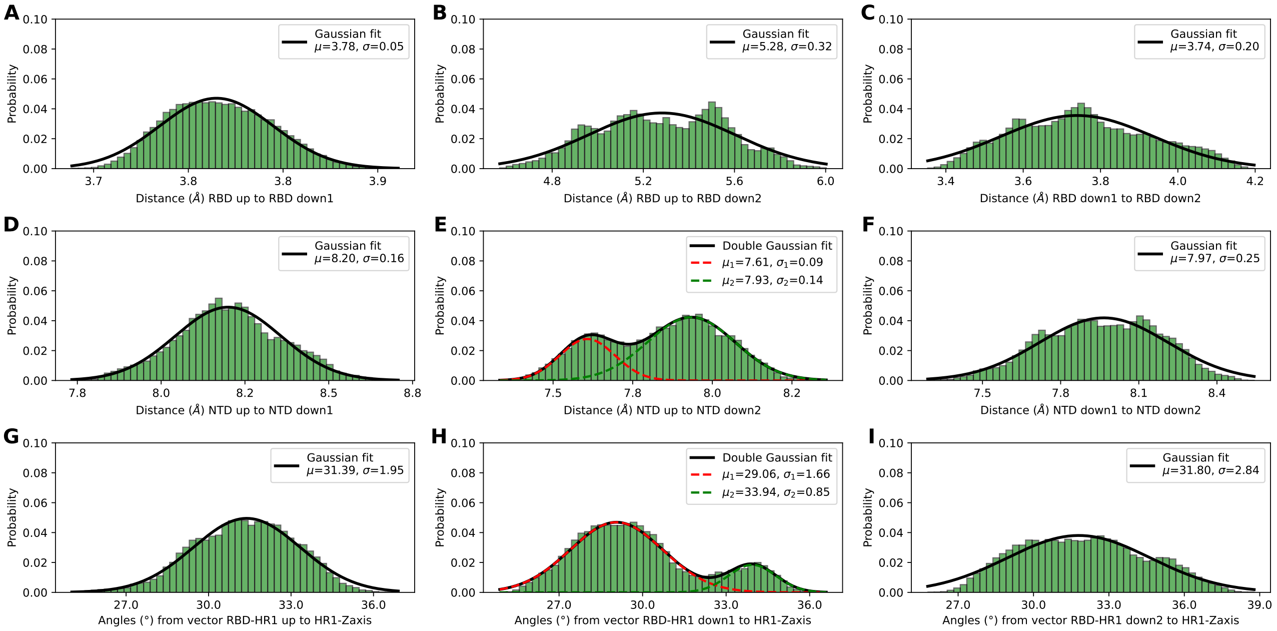

Figure S5: Gaussian distribution of the JN.1 variant for all calculated CVs. The black line indicates a fitting for a single Gaussian distribution while the dotted lines (red or green) for a double distribution.

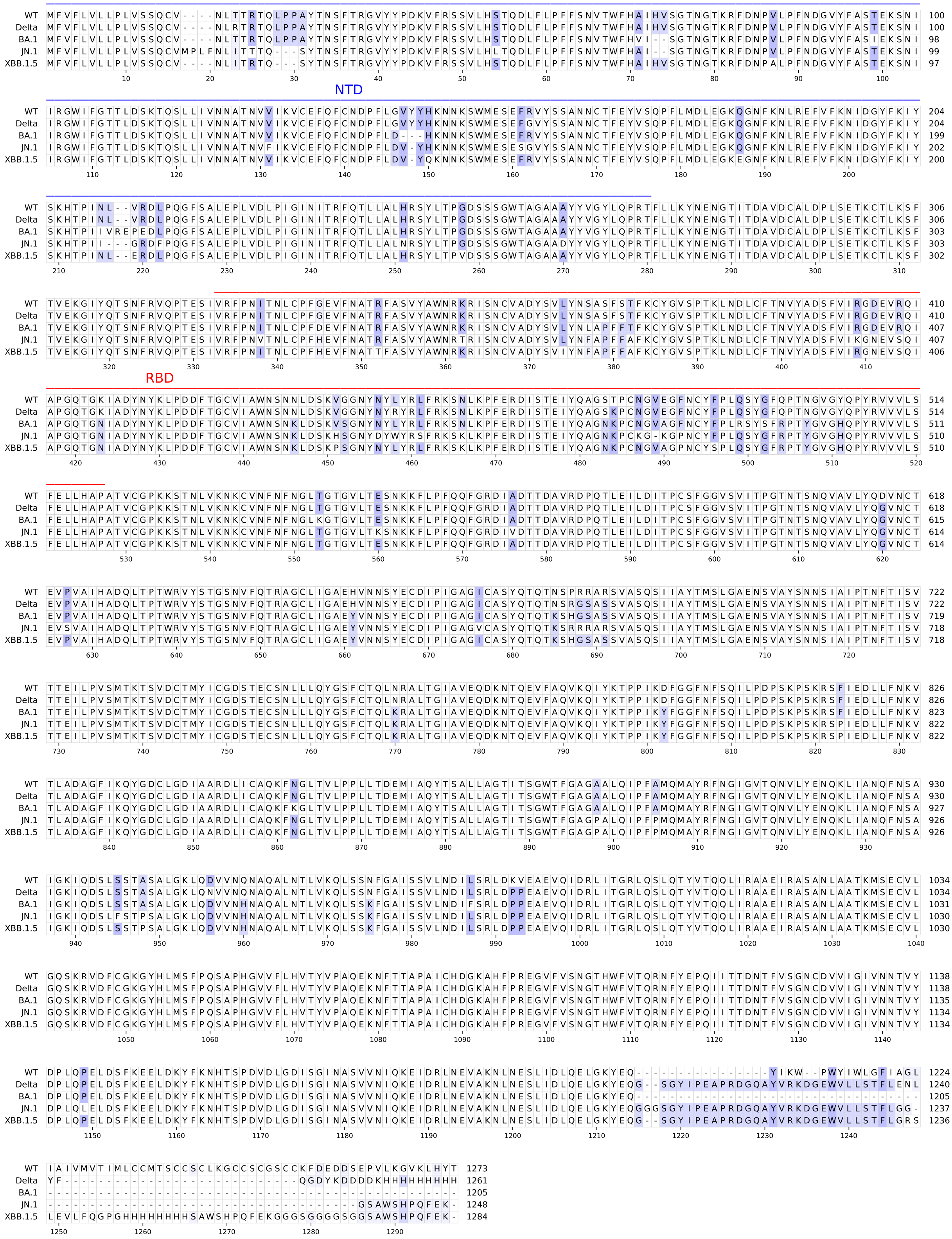

Figure S6: Alignment of the primary sequences of the WT S protein and its 4 variants. The WT sequence (PDB ID: 7CWL), as well as the sequences from Delta (PDB ID 7W92), BA.1 (PDB ID: 7XO5), JN.1 (PDB ID: 8Y5J), and XBB.1.5 (PDB ID: 8VKM) variants, were obtained in fasta format from the Protein Data Bank. Regions with at least one mutation are colored by identity (See pyMSAviz documentation). Note that insertions and deletions in the sequences of the variants modify the reading frame alignment.

Supplementary Tables

Table S1: Values of each CV (calculated between RBDs), σ, and χ^2 averages of the different states obtained by the probability distribution.

Collective Variables	Variants	States	μ	σ	χ^2
CV_1 (nm)	WT	1	4.24	0.11	0.04
		2	5.12	0.24	
	Delta	1	4.47	0.13	0.07
		2	5.44	0.32	
	BA.1	1	5.22	0.20	0.04
	XBB.1.5	1	4.77	0.12	0.09
	JN.1	1	3.78	0.05	0.03
CV_2 (nm)	WT	1	3.93	0.06	0.03
		2	4.36	0.12	
	Delta	1	4.19	0.14	0.03
		2	4.58	0.28	
	BA.1	1	4.10	0.09	0.23
	XBB.1.5	1	3.90	0.05	0.09
		2	4.04	0.06	
	JN.1	1	5.28	0.32	0.07

Table S2: Values of each CV (calculated between NTDs), σ, and χ^2 averages of the different states obtained by the probability distribution.

Collective Variables	Variants	States	μ	σ	χ^2
CV_3 (nm)	WT	1	3.42	0.10	0.14
		2	3.78	0.07	
	Delta	1	3.44	0.10	0.01
	BA.1	1	3.36	0.05	0.01
		2	3.56	0.12	
	XBB.1.5	1	3.46	0.07	0.02
	JN.1	1	3.74	0.20	0.05
CV_4 (nm)	WT	1	8.32	0.25	0.14
	Delta	1	8.57	0.27	0.04
	BA.1	1	7.79	0.18	0.09
	XBB.1.5	1	7.92	0.10	<0.01
		2	8.17	0.12	
	JN.1	1	8.20	0.16	0.02
CV_5 (nm)	WT	1	8.45	0.15	0.04
	Delta	1	8.73	0.24	0.04
	BA.1	1	7.58	0.12	0.01
		2	7.86	0.06	
		3	8.16	0.11	
	XBB.1.5	1	8.23	0.11	0.03
	JN.1	1	7.61	0.09	<0.01
		2	7.93	0.14	

Table S3: Values of each CV (calculated with angles of RBDs), σ, and χ^2 averages of the different states obtained by the probability distribution.

Collective Variables	Variants	States	μ	σ	χ^2
CV_7 (°)	WT	1	31.51	1.37	0.04
		2	37.00	2.00	
	Delta	1	31.01	4.44	0.01
		2	36.13	2.12	
	BA.1	1	32.28	1.37	0.02
		2	40.13	2.33	
	XBB.1.5	1	26.94	1.41	<0.01
		2	29.84	0.59	
	JN.1	1	31.39	1.95	0.02
CV_8 (°)	WT	1	26.32	2.61	0.05
	Delta	1	30.56	2.45	0.04
	BA.1	1	24.37	2.25	0.02
	XBB.1.5	1	23.94	1.38	0.17
	JN.1	1	29.06	1.66	0.01
		2	33.94	0.85	
CV_9 (°)	WT	1	27.17	1.77	0.25
		2	31.42	1.03	
	Delta	1	25.75	1.88	0.12
	BA.1	1	27.16	2.34	0.02
	XBB.1.5	1	25.03	1.03	<0.01
	JN.1	1	31.80	2.84	0.05

Table S4: Missing residues range remodeled into the chains of the S protein variants.

Delta			BA.1		
A	B	C	A	B	C
70-76<br>156-157<br>248-254<br>624-631<br>677-688<br>828-853	70-76<br>156-157<br>248-254<br>622-640<br>677-688<br>828-853	70-76<br>156-157<br>248-254<br>622-629<br>677-688<br>828-853	71-79<br>146-156<br>177-186<br>212-214<br>621-639<br>677-690<br>829-853	71-79<br>146-156<br>177-186<br>212-214<br>621-640<br>677-689<br>829-854	71-79<br>146-157<br>177-186<br>212-214<br>621-640<br>677-659<br>829-584
**XBB.1.5**			**JN.1**		
A	B	C	A	B	C
72-77<br>145-152<br>179-186<br>250-255<br>621-640<br>676-690<br>828-847	72-77<br>145-152<br>179-186<br>250-255<br>621-640<br>676-690<br>828-847	72-77<br>145-152<br>179-186<br>250-255<br>621-640<br>676-690<br>828-847	70-80<br>140-143<br>145-152<br>173-187<br>244-163<br>677-688<br>828-654	70-80<br>139-143<br>145-153<br>173-187<br>244-263<br>517-529<br>677-688<br>828-854	66-68<br>70-80<br>140-143<br>145-152<br>173-187<br>244-263<br>677-688<br>828-854

Table S5: High frequency NCs (freq > 60%) between the RBDs in the WT protein.

CV_1		CV_2		CV_3	
State 1<br>(NCs=0)	State 2<br>(NCs=0)	State 1<br>(NCs=6)	State 2<br>(NCs=4)	State 1<br>(NCs=2)	State 2<br>(NCs=8)
—	—	Specific NCs<br>F374—F486<br>F377—F486<br>Nonspecific NCs<br>S375—F486<br>T376—F486<br>S383—F456<br>T385—K458	Nonspecific NCs<br>S375—F486<br>F377—Y489<br>T385—K458<br>T385—F456	Nonspecific NCs<br>T415—P384<br>K417—Y369	Specific NCs<br>F456—A372<br>Nonspecific NCs<br>K417—A372<br>F456—Y369<br>F456—N370<br>R457—Y369<br>K458—Y369<br>K458—T385<br>K458—N388

Note: — ionic interactions, — polar interactions, — nonpolar interactions, — nonspecific interactions. * Mutated amino acids.

Table S6: High frequency NCs (freq > 60%) between the RBDs in the Delta variant.

CV_1		CV_2		CV_3
State 1<br>(NCs=0)	State 2<br>(NCs=0)	State 1<br>(NCs=9)	State 2<br>(NCs=10)	State 1<br>(NCs=5)
—	—	Specific NCs<br>Y369—N487<br>N370—S477<br>A372—F486<br>F374—F486<br>F377—F486<br>P384—F456<br>P384—Y489<br>T385—Y473<br>Nonspecific NC<br>S383—F456	Specific NCs<br>Y369—N487<br>N370—S477<br>F374—F486<br>F377—F486<br>Nonspecific NCs<br>Y369—G476<br>N370—K478*<br>S371—K478*<br>A372—K478*<br>P384—Y489<br>T385—A475	Specific NCs<br>T415—T385<br>Nonspecific NCs<br>D405—S375<br>R408—T376<br>T415—P384<br>K417—N370

Note: — ionic interactions, — polar interactions, — nonpolar interactions, — nonspecific interactions. * Mutated amino acids.

Table S7: High frequency NCs (freq > 60%) between the RBDs in the BA.1 variant.

CV_1	CV_2	CV_3	
State 1 (NCs=0)	State 1 (NCs=7)	State 1 (NCs=3)	State 2 (NCs=1)
—	Specific NC<br>F377—F486<br>D428—H505*<br>Nonspecific NCs<br>Y369—F486<br>S383—L455<br>S383—F456<br>P384—Y489<br>T385—F456	Specific NCs<br>Q414—T385<br>T415—T385<br>Nonspecific NCs<br>H505*—A372	Specific NC<br>T415—T385

Note: — ionic interactions, — polar interactions, — nonpolar interactions, — nonspecific interactions. * Mutated amino acids.

Table S8: High frequency NCs (freq > 60%) between the RBDs in the XBB.1.5 variant.

CV_1	CV_2		CV_3
State 1 (NCs=0)	State 1 (NCs=24)	State 2 (NCs=14)	State 1 (NCs=4)
—	Specific NCs<br>P373*—P486*<br>Y380—Q493<br>G381—L455<br>V382—L455<br>P384—F456<br>T385—Y473<br>D427—H505*<br>D428—H505*<br>Nonspecific NCs<br>Y369—A475<br>P373*—N487<br>F374—N487<br>F374—Y489<br>F377—Y489<br>C379—L455<br>C379—Q493<br>G381—N417*<br>G381—Y453<br>S383—L455<br>P384—Y473<br>T385—F456<br>T385—R457<br>G413—R498*<br>Q414—R498*<br>D427—Y501*	Specific NCs<br>F374—P486<br>F377—P486<br>P384—L455<br>T385—Y489<br>T385—Y473<br>Nonspecific NCs<br>S383—L455<br>S383—F456<br>P384—Y489<br>T385—F456<br>G413—R498*<br>D427—Y501*<br>D427—G502	Specific NCs<br>T415—T385<br>Nonspecific NCs<br>N405*—F374<br>N405*—F375*<br>H505*—F375*

Note: — ionic interactions, — polar interactions, — nonpolar interactions, — nonspecific interactions. * Mutated amino acids.

Table S9: High frequency NCs (freq > 60%) between the RBDs in the JN.1 variant.

CV_1	CV_2	CV_3
State 1 (NCs=0)	State 1 (NCs=24)	State 1 (NCs=0)
	Specific NCs	
	G416–P520	
	P485*–F374*	
	Y421–T522	
	T499–T415	
	S455*–S383	
	Y500*–Q414	
	F456–V382	
	Y500*–T415	
	Nonspecific NCs	
	N405*–D427	
	N405*–D428	
—	T415–A519	—
	N417*–G381	
	Y421–A521	
	Y421–P520	
	Y421–V382	
	S455*–V382	
	F456–S383	
	R457–V382	
	R457–T522	
	K458–T385	
	K458–T522	
	A475–T385	
	Y488–F377	
	H504*–G413	

Note: – ionic interactions, – polar interactions, – nonpolar interactions, – nonspecific interactions. * Mutated amino acids.

Table S10: High frequency NCs (freq > 60%) between an NTD and RBD in the WT protein.

CV_4	CV_5	CV_6
State 1 (NCs=4)	State 1 (NCs=6)	State 1 (NCs=1)
	Nonspecific NCs	
Nonspecific NCs	R357–F168	
F168–R357	R357–P230	
T167–R357	Y396–P230	Nonspecific NC
Y200–P521	H519–V42	P230–N394
P230–T523	A520–K41	
	P521–K41	

Note: – ionic interactions, – polar interactions, – nonpolar interactions, – nonspecific interactions. * Mutated amino acids.

Table S11: High frequency NCs (freq > 60%) between an NTD and RBD in the Delta variant.

CV_4	CV_5	CV_6
State 1<br>(NCs=0)	State 1<br>(NCs=3)	State 1<br>(NCs=3)
—	Nonspecific NCs<br>R357—P230<br>A520—K41<br>P521—K41	Nonspecific NCs<br>R357—P230<br>A520—K41<br>P521—K41

Note: — ionic interactions, — polar interactions, — nonpolar interactions, — nonspecific interactions. * Mutated amino acids.

Table S12: High frequency NCs (freq > 60%) between an NTD and RBD in the BA.1 variant.

CV_4		CV_5		CV_6	
State 1<br>(NCs=2)	State 1<br>(NCs=21)	State 2<br>(NCs=26)	State 3<br>(NCs=4)	State 1<br>(NCs=7)	State 2<br>(NCs=6)
Specific NC<br>P521—P230<br>Nonspecific NC<br>S359—E167	Specific NCs<br>R357—E167<br>F464—G232<br>F464—I233<br>Nonspecific NCs<br>R355—Q116<br>R355—T165<br>R357—F166<br>R357—Y168<br>N394—P230<br>Y396—P230<br>Y396—I231<br>Y396—G232<br>P463—N234<br>F464—N234<br>E465—N234<br>R466—T115<br>R466—Q116<br>R466—I233<br>R466—N234<br>D467—T115<br>I468—K114<br>H519—V45	Specific NCs<br>R357—E167<br>F464—G197<br>F464—G232<br>F464—I233<br>Nonspecific NCs<br>R355—Q116<br>R355—F166<br>R355—G232<br>K356—F166<br>R357—F166<br>R357—T165<br>R357—Y168<br>N394—D228<br>N394—P230<br>Y396—I231<br>Y396—G232<br>Y396—P230<br>P426—D196<br>F464—N234<br>E465—N234<br>R466—Q116<br>R466—I233<br>R466—T115<br>R466—N234<br>D467—T115<br>I468—K114<br>H519—V45	Nonspecific NCs<br>K44—A520<br>K44—P521<br>V45—H519<br>P230—R357	Specific NCs<br>G197—F464<br>G197—P463<br>Nonspecific NCs<br>K44—A520<br>K44—P521<br>V45—H519<br>Q116—I468<br>P230—R357	Specific NC<br>G197—P463<br>Nonspecific NCs<br>K44—A520<br>K44—P521<br>V45—H519<br>Q116—I468<br>P230—R357

Note: — ionic interactions, — polar interactions, — nonpolar interactions, — nonspecific interactions. * Mutated amino acids.

Table S13: High frequency NCs (freq > 60%) between an NTD and RBD in the XBB.1.5 variant.

CV$_4$		CV$_5$	CV$_6$	
State 1 (NCs=10)	State 2 (NCs=12)	State 1 (NCs=9)	State 1 (NCs=8)	State 2 (NCs=13)
Specific NCs<br>A520–G232<br>P521–G199<br>P521–G232<br>P521–P230<br>Nonspecific NCs<br>R357–C166<br>N360–E169<br>N360–F168<br>P521–Y200<br>T523–P230<br>A522–Y200	Specific NCs<br>N360–Y170<br>A520–G232<br>P521–G199<br>P521–G232<br>P521–P230<br>Nonspecific NCs<br>R357–C166<br>N360–F168<br>N360–E169<br>P521–Y200<br>A522–Y200<br>T523–F168<br>T523–P230	Specific NCs<br>N394–Y200<br>F464–G232<br>Nonspecific NCs<br>R357–F168<br>R357–P230<br>Y396–P230<br>K462–N234<br>F464–N234<br>E465–N234<br>P521–K42	Specific NCs<br>Y200–N394<br>Y200–Y396<br>Nonspecific NCs<br>K42–P521<br>T167–R357<br>F168–R357<br>P230–R357<br>P230–Y396<br>N234–E465	Specific NCs<br>G199–F464<br>Y200–N394<br>Y200–Y396<br>Nonspecific NCs<br>K42–P521<br>F168–R357<br>D198–F464<br>G199–Y396<br>P230–R357<br>P230–Y396<br>G232–R355<br>G232–Y396<br>N234–E465<br>N234–F464

Note: – ionic interactions, – polar interactions, – nonpolar interactions, – nonspecific interactions. * Mutated amino acids.

Table S14: High frequency NCs (freq > 60%) between an NTD and RBD in the JN.1 variant.

CV$_4$	CV$_5$		CV$_6$
State 1 (NCs=0)	State 1 (NCs=14)	State 2 (NCs=13)	State 1 (NCs=10)
—	Specific NC<br>G200–F464<br>Nonspecific NCs<br>K45–P520<br>Q117–R466<br>F169–R357<br>D199–P463<br>D199–F464<br>P230–R357<br>P230–N394<br>P230–Y396<br>I231–R355<br>G232–R355<br>G232–R466<br>N234–D467<br>N234–E465	Specific NC<br>G200–F464<br>Nonspecific NCs<br>K45–P520<br>F169–R357<br>D199–P463<br>D199–F464<br>P230–R357<br>P230–Y396<br>I231–R355<br>G232–R355<br>G232–R466<br>I233–R466<br>N234–E465<br>N234–R466	Specific NC<br>Y396–Y201<br>Nonspecific NCs<br>R355–G232<br>R357–P230<br>V395–Y201<br>Y396–G200<br>Y396–P230<br>Y396–G232<br>E465–N234<br>I468–T116<br>H518–K45

Note: – ionic interactions, – polar interactions, – nonpolar interactions, – nonspecific interactions. * Mutated amino acids.

Table S15: High frequency NCs (freq > 60%) for the CVs calculated with angles in the WT protein.

NCs between two RBDs				
CV$_7$		CV$_8$	CV$_9$	
State 1<br>(NCs=0)	State 2<br>(NCs=0)	State 1<br>(NCs=7)	State 1<br>(NCs=0)	State 2<br>(NCs=6)
—	—	Specific NCs<br>F374–F486<br>F377–F486<br>Nonspecific NCs<br>S375–F486<br>T376–F486<br>S383–F456<br>T385–F456<br>T385–K458	—	Nonspecific NCs<br>T415–F377<br>T415–P384<br>K417–Y369<br>F456–Y369<br>F456–N370<br>K458–N388
NCs between an RBD and an NTD				
CV$_7$		CV$_8$	CV$_9$	
State 1<br>(NCs=11)	State 2<br>(NCs=7)	State 1<br>(NCs=6)	State 1<br>(NCs=3)	State 2<br>(NCs=5)
Nonspecific NCs<br>T167–R355<br>T167–K356<br>T167–R357<br>F168–R357<br>F168–S359<br>D228–N360<br>L229–N360<br>P230–S359<br>P230–T523<br>I231–R357<br>G232–R357	Specific NCs<br>P230–P521<br>P230–A520<br>G232–L518<br>Nonspecific NCs<br>C166–R357<br>T167–R357<br>Y200–P521<br>P230–T523	Nonspecific NCs<br>R357–F168<br>R357–P230<br>Y396–P230<br>H519–V42<br>A520–K41<br>P521–K41	Nonspecific NCs<br>Y200–A520<br>D228–P521<br>P230–T523	Nonspecific NCs<br>P230–N394<br>P230–Y396<br>G232–Y396<br>N234–E465<br>N234–R466

Note: – ionic interactions, – polar interactions, – nonpolar interactions, – nonspecific interactions. * Mutated amino acids.

Table S16: High frequency NCs (freq > 60%) for the CVs calculated with angles in the Delta variant.

NCs between two RBDs			
CV_7		CV_8	CV_9
State 1 (NCs=0)	State 2 (NCs=0)	State 1 (NCs=6)	State 1 (NCs=4)
—	—	Specific NCs<br>Y369—N487<br>N370—S477<br>F374—F486<br>F377—F486<br>P384—F456<br>Nonspecific NCs<br>P384—Y489	Specific NCs<br>T415—T385<br>Nonspecific NCs<br>R408—T376<br>T415—P384<br>K417—N370
**NCs between an RBD and an NTD**			
CV_7		CV_8	CV_9
State 1 (NCs=0)	State 2 (NCs=0)	State 1 (NCs=3)	State 1 (NCs=1)
—	—	Nonspecific NCs<br>R357—P230<br>A520—K41<br>P521—K41	Nonspecific NCs<br>P230—R357

Note: — ionic interactions, — polar interactions, — nonpolar interactions, — nonspecific interactions. * Mutated amino acids.

Table S17: High frequency NCs (freq > 60%) for the CVs calculated with angles in the BA.1 variant.

NCs between two RBDs			
CV_7		CV_8	CV_9
State 1<br>(NCs=0)	State 2<br>(NCs=0)	State 1<br>(NCs=9)	State 1<br>(NCs=1)
—	—	Specific NCs<br>F377–F486<br>P384–L455<br>T385–Y489<br>D428–H505*<br>Nonspecific NCs<br>Y369–F486<br>S383–L455<br>S383–F456<br>P384–Y489<br>T385–F456	Specific NC<br>T415–T385
**NCs between an RBD and an NTD**			
CV_7		CV_8	CV_9
State 1<br>(NCs=21)	State 2<br>(NCs=1)	State 1<br>(NCs=21)	State 1<br>(NCs=4)
Specific NCs<br>L335–V169<br>P337–V169<br>N354–N163<br>R357–E167<br>N360–Y168<br>R466–E133<br>P521–P230<br>Nonspecific NCs<br>L335–S170<br>L335–Q171<br>P337–E167<br>R355–T165<br>K356–T165<br>R357–T165<br>R357–F166<br>S359–F166<br>S359–E167<br>N360–P230<br>R466–N162<br>R466–N163<br>I468–N163<br>T523–P230	Specific NC<br>P521–P230	Specific NCs<br>R357–E167<br>F464–G232<br>F464–I233<br>Nonspecific NCs<br>R355–Q116<br>R355–T165<br>R357–F166<br>R357–Y168<br>N394–P230<br>Y396–P230<br>Y396–I231<br>Y396–G232<br>P426–D196<br>F464–N234<br>E465–N234<br>R466–T115<br>R466–Q116<br>R466–I233<br>R466–N234<br>D467–T115<br>I468–K114<br>H519–V45	Nonspecific NCs<br>K44–A520<br>K44–P521<br>V45–H519<br>P230–R357

Note: – ionic interactions, – polar interactions, – nonpolar interactions, – nonspecific interactions. * Mutated amino acids.

Table S18: High frequency NCs (freq > 60%) for the CVs calculated with angles in the XBB.1.5 variant.

NCs between two RBDs			
CV_7		CV_8	CV_9
State 1<br>(NCs=0)	State 2<br>(NCs=0)	State 1<br>(NCs=17)	State 1<br>(NCs=3)
—	—	Specific NCs<br>P373*–P486*<br>F374–P486*<br>P384–F456<br>P384–L455<br>T385–Y473<br>D427–H505*<br>D428–H505*<br>Nonspecific NCs<br>F377–Y489<br>C379–L455<br>C379–Q493<br>G381–Y453<br>S383–L455<br>T385–F456<br>G413–R498*<br>G413–Y501*<br>Q414–R498*<br>D427–Y501*	Specific NC<br>T415–T385<br>Nonspecific NCs<br>N405*–F374<br>N405*–F375*
**NCs between an RBD and an NTD**			
CV_7		CV_8	CV_9
State 1<br>(NCs=11)	State 2<br>(NCs=12)	State 1<br>(NCs=9)	State 1<br>(NCs=9)
Specific NCs<br>N360–Y170<br>A520–G232<br>P521–G199<br>P521–P230<br>P521–G232<br>Nonspecific NCs<br>R357–C166<br>N360–F168<br>N360–E169<br>P521–Y200<br>A522–Y200<br>T523–P230	Specific NCs<br>S359–T167<br>A520–G232<br>P521–G199<br>P521–P230<br>P521–G232<br>Nonspecific NCs<br>R357–C166<br>N360–F168<br>N360–E169<br>P521–Y200<br>A522–Y200<br>T523–P230<br>T523–F168	Specific NC<br>N394–Y200<br>Nonspecific NCs<br>R357–F168<br>R357–P230<br>Y396–P230<br>Y396–G232<br>K462–N234<br>F464–N234<br>E465–N234<br>P521–K42	Specific NC<br>Y200–N394<br>Y200–Y396<br>Nonspecific NCs<br>F168–R357<br>K42–P521<br>P230–R357<br>P230–Y396<br>G232–R355<br>G232–Y396<br>N234–E465

Note: – ionic interactions, – polar interactions, – nonpolar interactions, – nonspecific interactions. * Mutated amino acids.

Table S19: High frequency NCs (freq > 60%) for the CVs calculated with angles in the JN.1 variant.

NCs between two RBDs			
CV$_7$	CV$_8$		CV$_9$
State 1 (NCs=0)	State 1 (NCs=22)	State 2 (NCs=36)	State 1 (NCs=0)
—	Specific NCs	Specific NCs	—
	G416—P520	G416—P520	
	Y421—T522	Y421—T522	
	S455—S383	S455—S383	
	F456—V382	F456—V382	
	T499—T415	F456—P384	
	Y500*—Q414	Y473—T385	
	Y500*—T415	P485*—F374	
	H504*—D427	N486—T385	
	Nonspecific NCs	Y488—T385	
	N405*—D428	T499—T415	
	T415—A519	Y500*—Q414	
	N417*—G381	Y500*—T415	
	Y421—V382	Nonspecific NCs	
	Y421—P520	N405*—D427	
	Y421—A521	N405*—D428	
	S455*—V382	T415—A519	
	F456—S383	T415—P520	
	R457—V382	N417*—G381	
	R457—T522	Y421—V382	
	K458—T522	Y421—P520	
	Y488—F377	S455*—V382	
	R497*—T415	F456—S383	
	H504*—G413	F456—T385	
		R457—V382	
		R457—T522	
		K458—T385	
		K458—T522	
		A475—T385	
		A475—K386	
		K483*—Y369	
		K483*—N370	
		G484—Y369	
		P485*—Y369	
		N486—K386	
		Y488—F377	
		H504*—G413	
		H504*—Q414	

NCs between an RBD and an NTD			
CV$_7$	CV$_8$		CV$_9$
State 1 (NCs=0)	State 1 (NCs=15)	State 2 (NCs=2)	State 1 (NCs=10)
—	Specific NCs	Nonspecific NCs	Specific NCs
	G200—P463	K45—P520	Y396—Y201
	G200—F464	P230—R357	Nonspecific NCs
	Nonspecific NCs		R355—G232
	K45—P520		R357—P230
	F169—R357		Y396—G200
	D199—P463		Y396—P230
	D199—F464		Y396—G232
	P230—R357		E465—N234
	P230—Y396		I468—K115
	I231—R355		I468—T116
	G232—R355		H518—K45
	G232—R466		
	I233—R466		
	N234—E465		
	N234—R466		
	N234—D467		

Note: — ionic interactions, — polar interactions, — nonpolar interactions, — nonspecific interactions. * Mutated amino acids.
